# Supplementary material for: Persistence of Metabolomic Changes in Patients during Post-COVID Phase: A Prospective, Observational Study
Source: Metabolites. 2022 Jul 13;12(7):641. doi: 10.3390/metabo12070641 (PMC9321209; doi:10.3390/metabo12070641)
Supplement: Supplementary file 1 [file metabolites-12-00641-s001.zip › Supplement S1.pdf]

**Supplement S1**

**PLS-DA VIP scores for the top 15 most important metabolites (including lipoprotein fraction not meeting the criteria of metabolite) in patients with COVID-19 disease at various sampling time for all three evaluated time points in patients with controls, as input variables were used relative concentrations of metabolites in blood plasma, metabolites ordered by VIP score for component1**

| | Comp.1 | Comp.2 | Comp. 3 | Comp.4 | Comp.5 |
|---|---|---|---|---|---|
| lipoproteins | 4.0326 | 3.9969 | 3.9613 | 3.9143 | 3.8618 |
| glucose | 1.5436 | 1.5352 | 1.5282 | 1.5626 | 1.6152 |
| alanine | 1.3407 | 1.3454 | 1.3394 | 1.3292 | 1.3082 |
| glutamine | 0.83501 | 0.83543 | 0.82773 | 0.84025 | 0.83143 |
| lactate | 0.82468 | 0.86773 | 0.98161 | 1.0017 | 0.99007 |
| 3-hydroxy-butyrate | 0.68815 | 0.69736 | 0.7043 | 0.69529 | 0.76874 |
| proline | 0.52723 | 0.52228 | 0.51735 | 0.51188 | 0.50433 |
| leucine | 0.42011 | 0.55035 | 0.57522 | 0.56789 | 0.62071 |
| pyruvate | 0.29886 | 0.3304 | 0.34726 | 0.37396 | 0.37281 |
| phenylalanine | 0.28886 | 0.3692 | 0.42934 | 0.48519 | 0.49484 |
| acetate | 0.20287 | 0.20248 | 0.20132 | 0.20262 | 0.20768 |
| citrate | 0.1223 | 0.14493 | 0.16657 | 0.18452 | 0.1944 |
| histidine | 0.099847 | 0.098934 | 0.098216 | 0.099839 | 0.10409 |
| isoleucine | 0.074585 | 0.076456 | 0.076502 | 0.089398 | 0.10706 |
| creatine | 0.069512 | 0.070573 | 0.084169 | 0.10703 | 0.12831 |
| lysine | 0.0413 | 0.10076 | 0.10001 | 0.14137 | 0.15355 |
| creatinine | 0.029174 | 0.028941 | 0.038152 | 0.080362 | 0.1079 |
| ketoleucine | 0.018621 | 0.043931 | 0.050523 | 0.056701 | 0.055812 |
| ketovaline | 0.017722 | 0.045458 | 0.059331 | 0.073259 | 0.073657 |
| valine | 0.011758 | 0.033175 | 0.14348 | 0.41944 | 0.5619 |

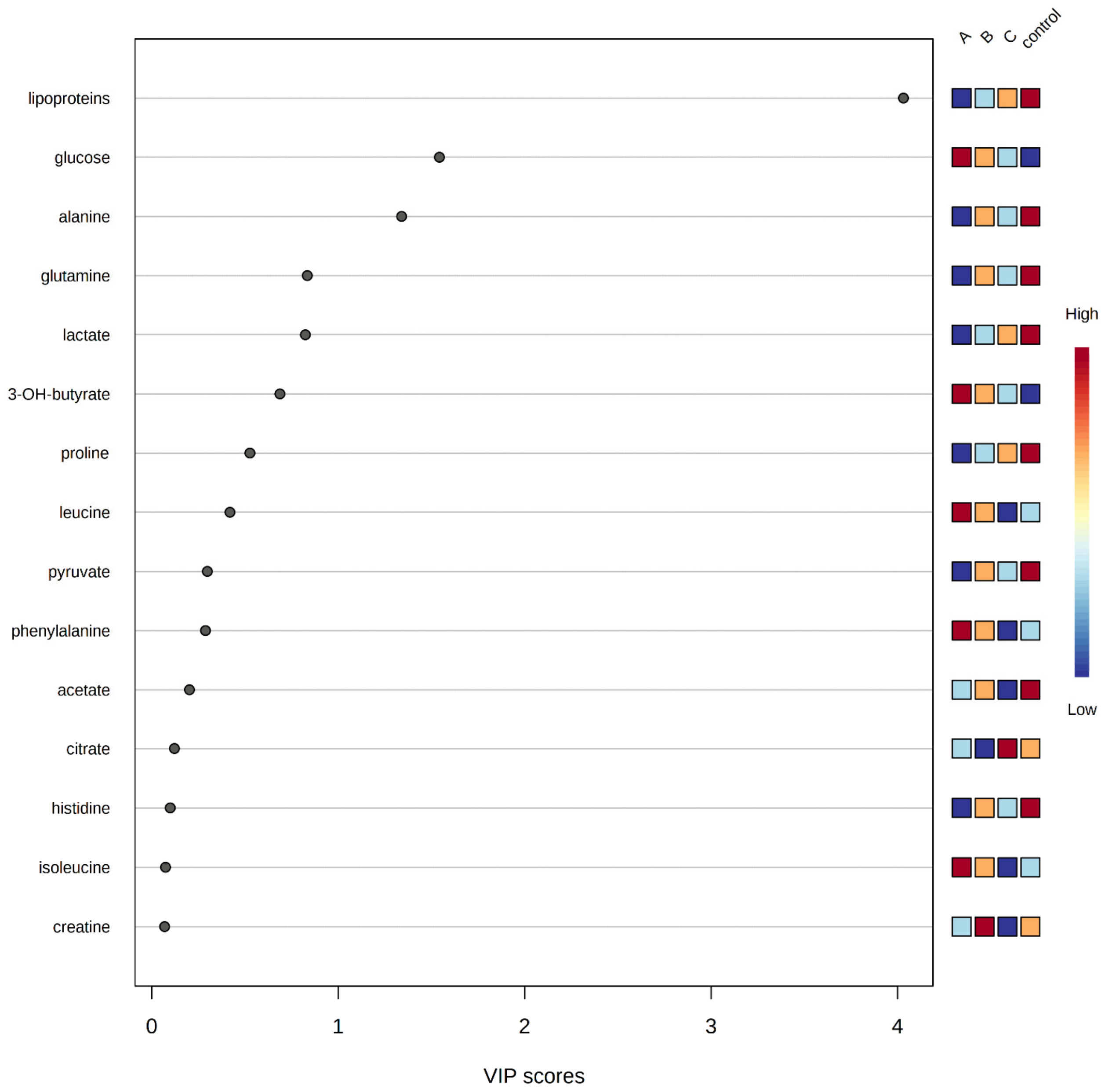

A
B
C
control
lipoproteins
glucose
alanine
glutamine
lactate
3-OH-butyrate
proline
leucine
pyruvate
phenylalanine
acetate
citrate
histidine
isoleucine
creatine
High
Low
0
1
2
3
4
VIP scores

**PLS-DA VIP scores for the top 15 most important metabolites (including lipoprotein fraction not meeting the criteria of metabolite) in patients with COVID-19 disease at sampling time C – in average 42 days after hospitalization and controls, as input variables were used relative concentrations of metabolites in blood plasma, analysis ran in metaboanalyst 5.0**

| | Comp.1 | Comp.2 | Comp. 3 | Comp.4 | Comp.5 |
|---|---|---|---|---|---|
| lipoproteins | 4.1577 | 4.0562 | 3.9982 | 3.9684 | 3.9363 |
| glutamine | 1.506 | 1.6481 | 1.6569 | 1.6437 | 1.651 |
| alanine | 1.2089 | 1.2015 | 1.2009 | 1.1949 | 1.185 |
| leucine | 0.76242 | 0.74531 | 0.73779 | 0.73575 | 0.74719 |
| glucose | 0.58015 | 0.7946 | 0.975 | 0.98837 | 0.9856 |
| lactate | 0.48237 | 0.47678 | 0.54427 | 0.71878 | 0.76194 |
| pyruvate | 0.4684 | 0.48077 | 0.47751 | 0.47996 | 0.48506 |
| valine | 0.39592 | 0.42059 | 0.41447 | 0.42139 | 0.46993 |
| proline | 0.38693 | 0.43053 | 0.45727 | 0.46006 | 0.47007 |
| lysine | 0.35696 | 0.36603 | 0.3785 | 0.37731 | 0.3752 |
| acetate | 0.34335 | 0.381 | 0.3965 | 0.41187 | 0.45321 |
| histidine | 0.16923 | 0.1717 | 0.17534 | 0.17592 | 0.17546 |
| phenylalanine | 0.13692 | 0.14584 | 0.15272 | 0.1543 | 0.15992 |
| creatine | 0.051207 | 0.10234 | 0.13285 | 0.14768 | 0.17929 |
| tyrosine | 0.050995 | 0.0522 | 0.055409 | 0.056972 | 0.059412 |
| ketoleucine | 0.047754 | 0.057661 | 0.056815 | 0.059418 | 0.068921 |
| citrate | 0.043144 | 0.06224 | 0.065622 | 0.075247 | 0.10464 |
| ketovaline | 0.042676 | 0.059605 | 0.059565 | 0.060357 | 0.066535 |
| 3-hydroxy-butyrate | 0.039289 | 0.038682 | 0.038972 | 0.038732 | 0.038937 |
| creatinine | 0.034986 | 0.14619 | 0.19039 | 0.2389 | 0.34733 |

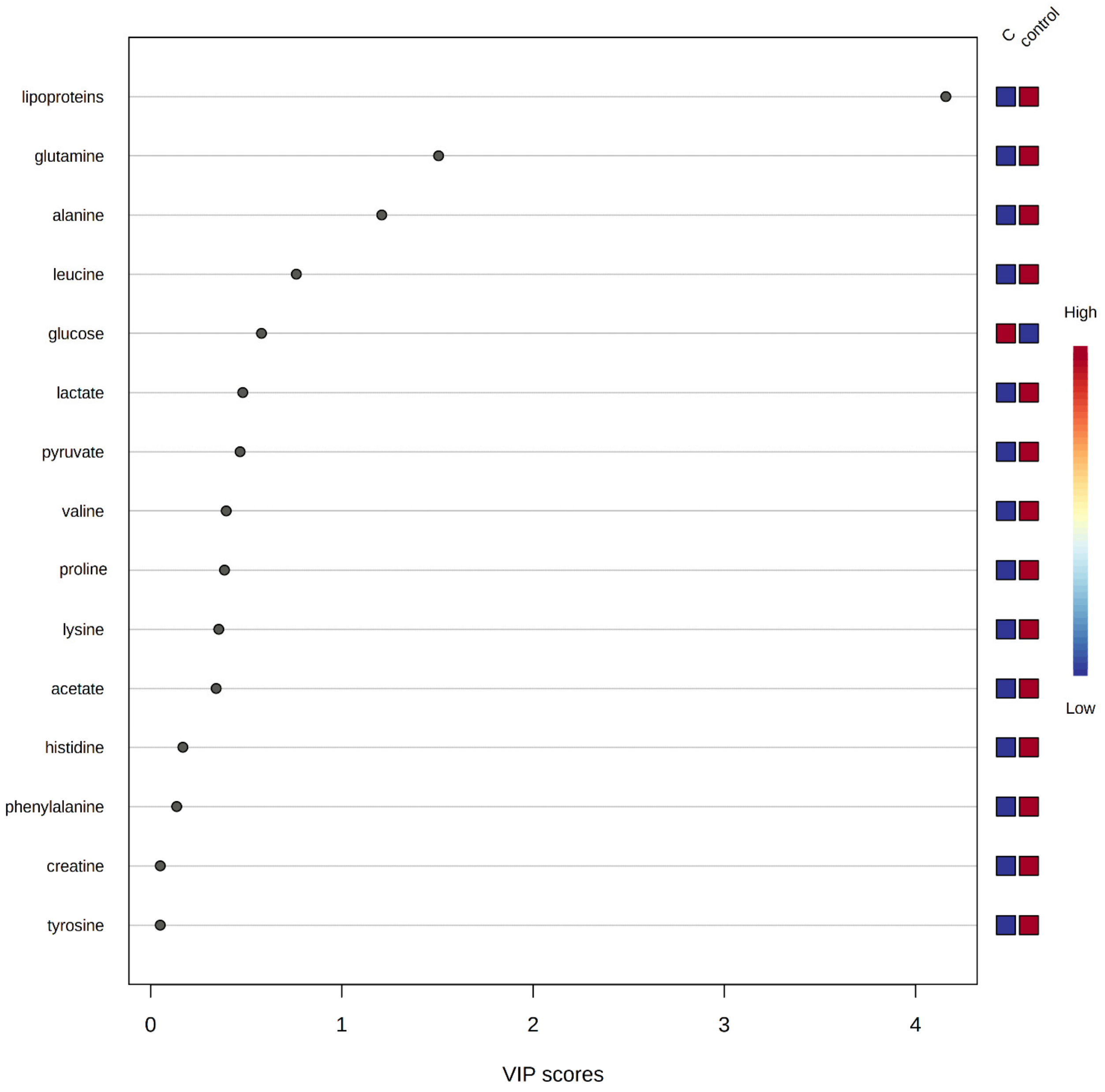
C
control
lipoproteins
glutamine
alanine
leucine
glucose
lactate
pyruvate
valine
proline
lysine
acetate
histidine
phenylalanine
creatine
tyrosine
High
Low
0
1
2
3
4
VIP scores
