# Supplementary material for: Persistence of Metabolomic Changes in Patients during Post-COVID Phase: A Prospective, Observational Study
Source: Metabolites. 2022 Jul 13;12(7):641. doi: 10.3390/metabo12070641 (PMC9321209; doi:10.3390/metabo12070641)

Supplement S2

NMR spectra – regions for the most interesting metabolites, comparison for: A-red, B-blue, C-yellow, control-green, the chemical shift values are listed in the Table S1.

Region of BCKAs (leucine, isoleucine, valine), ketovaline and ketosioleucine

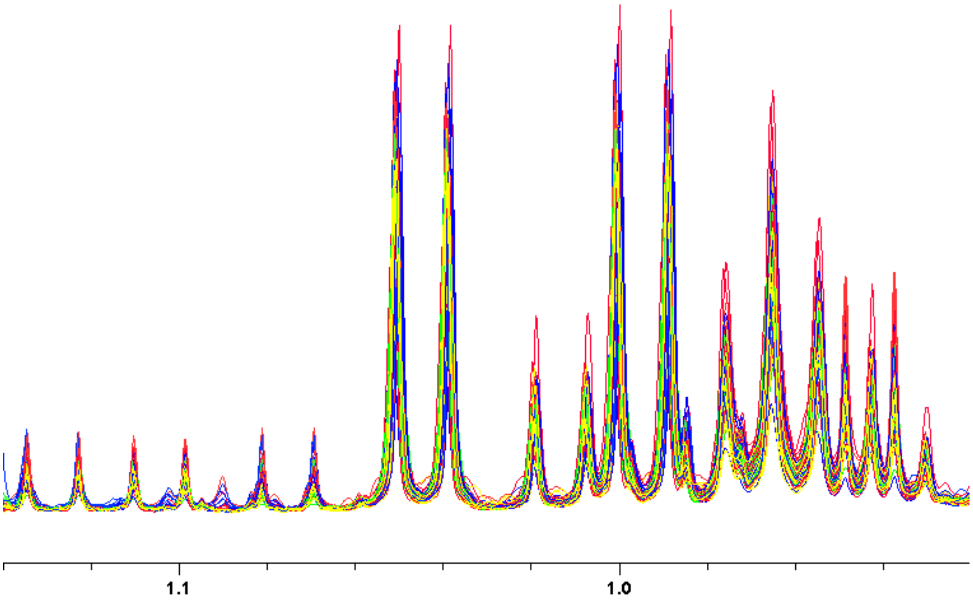

Region of : acetone (not evaluated), 3-hydroxybutyrate, pyruvate, succinate and glutamine

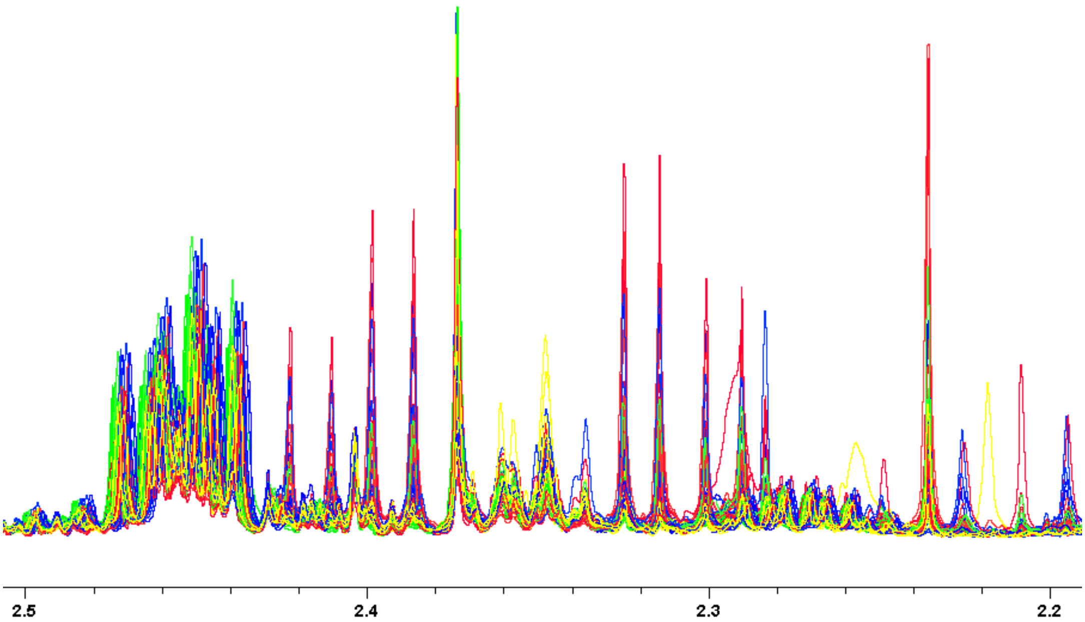

Regions of glucose and creatine

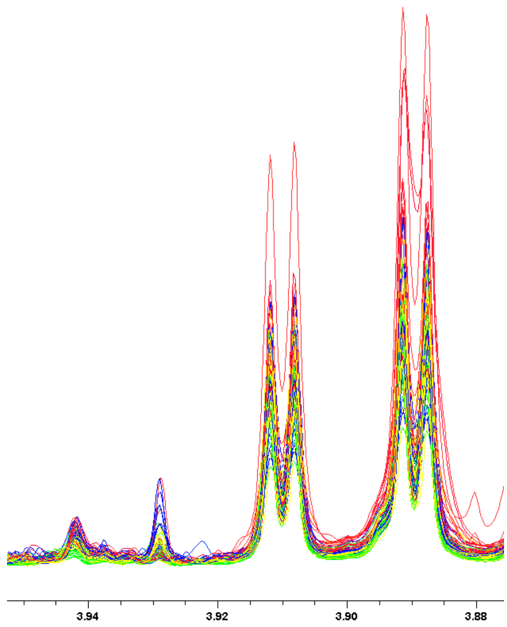

Region of lipoprotein fraction

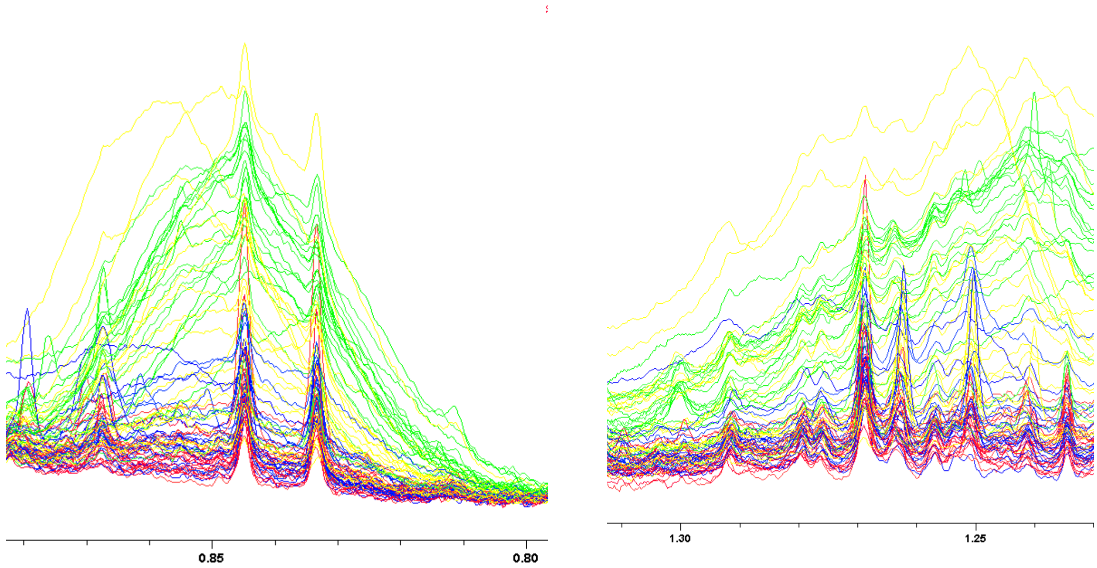

Supplement: Supplementary file 1 [file metabolites-12-00641-s001.zip › Supplement S2.pdf]
