# Supplementary material for: Persistence of Metabolomic Changes in Patients during Post-COVID Phase: A Prospective, Observational Study
Source: Metabolites. 2022 Jul 13;12(7):641. doi: 10.3390/metabo12070641 (PMC9321209; doi:10.3390/metabo12070641)

Supplement S3

Correlation heatmap for the relative concentrations of plasma metabolites, including data from patients in sampling poins A, B, C) and controls, evaluated by Pearsons'correlation

The plasma levels of branched chain amino acids (leucine, isoleucine and valine) as well as their ketoacids (2-ketoleucine, 2-ketoisoleucine and 2-ketovaline) are strongly related to each other, as they share common metabolomics pathways. There is also obvious that a ketone bodies representative, 3-hydroxybutyrate oppositely correlates with glucose, as they are competitive energy sources. Oposite correlation is visible also for 3-hydroxybutyrate and lipoproteins which are in the substrate-product relation.

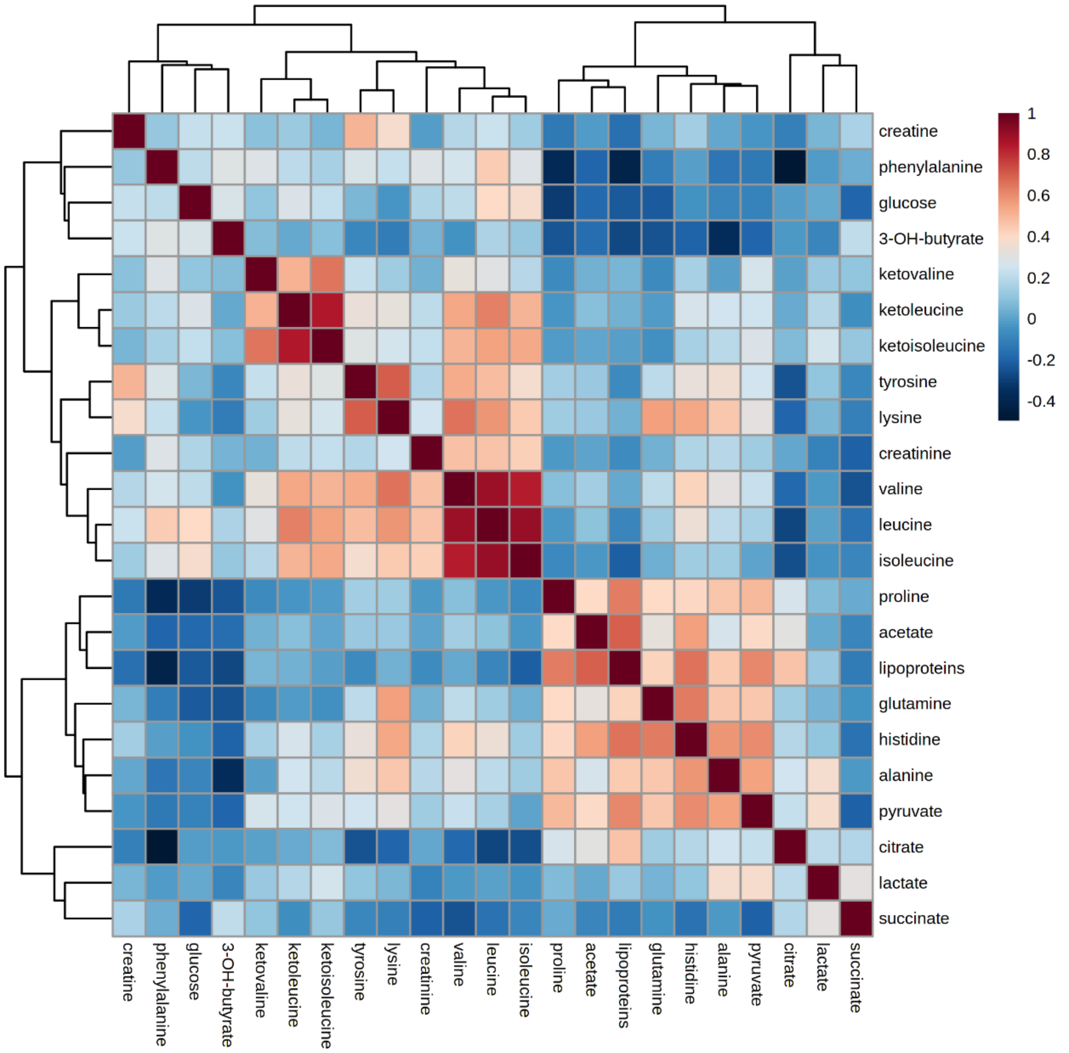

Supplement: Supplementary file 1 [file metabolites-12-00641-s001.zip › Supplement S3.pdf]
