# Supplementary material for: Persistence of Metabolomic Changes in Patients during Post-COVID Phase: A Prospective, Observational Study
Source: Metabolites. 2022 Jul 13;12(7):641. doi: 10.3390/metabo12070641 (PMC9321209; doi:10.3390/metabo12070641)
Supplement: Supplementary file 1 [file metabolites-12-00641-s001.zip › Table S1.pdf]

Table S1. Chemical shifts (in ppm), J couplings (in Hz) and multiplicities (s - singlet, d - doublet, t - triplet, q - quartet, m - multiplet, dd - doublet of doublets, dq - doublet of quartets) for the pool of metabolites identified in blood plasma. Signals marked with # were not suitable for quantitative analyzes.

metabolite	NMR peak assignment, confirmed by jres and cosy
# threonine	1.34 (d, J= 6.5), 3.56 (d; J= 4.9), 4.24 (dq, J= 4.9, 6.6)
# tryptophan	7.21 (t, J= 8.59), 7.30 (td, J= 7.3, 1.1,), 7.33 (s), 7.56 (d, J = 8.1), 7.74 (d; J = 8.0)
2-oxoisovalerate (2-ketovaline)	1.11 (d; J= 7.1), 3.01(m)
2-oxoisocapronate (2-ketoleucine)	0.94 (d; J= 6.6), 2.11 (m), 2.61 (d; J= 7.0)
3-hydroxybutyrate	1.20 (d; J= 6.23 Hz), 2.31 (m, J= 14.4, 6.2), 2.41 (m, J= 14.4, 7.2), 4.16 (dt, J= 7.2, 6.5)
3-methyl-2-oxo-valerate (2-ketoisoleucine)	0.90 (t; J=7.5), 1.10 (d; J= 6.7), 1.46(m), 1.70(m), 2.93(m)
acetate	1.92 (s)
alanine	1.48 (d; J=7.3), 3.78 (q, J= 7.2)
citrate	2.54 (d; J= 15.1), 2.67 (d; J= 15.1)
creatine	3.04 (s), 3.94 (s)
creatinine	3.05 (s), 4.07 (s)
glucose	3.24 (m), 3.40 (t, J=9.4), 3.41(m) 3.46 (m), 3.52 (t, J= 9.18), 3.78 (m), 3.84 (m), 3.89 (dd, J = 10.8, 9.8), 4.64 (d, J= 7.6) , 5.23 (d, J= 1.6)
glutamine	2.12 (m), 2.15 (m), 2.44 (m), 2.48 (m), 3.77 (t, J=6.2)
histidine	7.07 (d, J= 1.7), 7.80 (s)
isoleucine	0.94 (t; J= 7.5), 1.01 (d; J= 7.0), 3.68 (d; J= 4.2)
lactate	1.33 (d; J= 7.0), 4.12 (q; J= 6.9)
leucine	0.96 (d; J= 6.2), 0.97 (d; J= 6.1), 1.68 (m), 1.72 (m), 1.75(m),
lipoprotein fraction	0.82-0.93 (m), 1.20-1.37 (m)
lysine	1.44 (m), 1.51 (m), 1.71(m), 1.89 (m), 3.02(t, J= 7.54), 3.74(t, J= 8.1)
phenylalanine	3.13 (m), 3.28 (m), 7.34 (d; J= 7.5), 7.38 (t; J= 7.4), 7.44 (t, J= 7.6)
proline	1.99 (m), 1.99 (m), 2.05(m), 2.34 (m), 3.32(m), 3.41(m), 4.13 (dd, J= 6.37, 8.71)
pyruvate	2.38 (s)
tyrosine	3.20 (m), 3.93 (m), 6.91 (d; J= 8.5), 7.20 (d; J= 8.5)
valine	0.99 (d; J= 7.1), 1.04 (d; J= 7.1), 2.27 (m), 3.61 (d; J= 4.5)

* Coupling constants were read out manually in Topspin 3.6.1 using 1D cpmg or 2D Jres spectra and rounded to the one decimal place.
